# Fast acquisition abdominal MRI study for the investigation of suspected acute appendicitis in paediatric patients

**DOI:** 10.1186/s13244-020-00882-7

**Published:** 2020-06-16

**Authors:** Karl James, Patrick Duffy, Richard G. Kavanagh, Brian W. Carey, Stephen Power, David Ryan, Stella Joyce, Aoife Feeley, Peter Murphy, Emmet Andrews, Mark F. McEntee, Michael Moore, Conor Bogue, Michael M. Maher, Owen J. O’ Connor

**Affiliations:** 1 Department of Radiology, Cork University Hospital, Cork, Ireland; 2 Department of Radiology, University College Cork, Cork, Ireland; 3 School of Medicine, University College Cork, Cork, Ireland; 4 PET/CT-MRI Unit, Cork University Hospital, Cork, Ireland; 5 Department of Surgery, Cork University Hospital, Cork, Ireland; 6 Department of Radiography, University College Cork, Cork, Ireland

**Keywords:** Acute appendicitis, Magnetic resonance imaging, Ultrasound, Paediatric

## Abstract

**Objectives:**

To assess the diagnostic accuracy of fast acquisition MRI in suspected cases of paediatric appendicitis presenting to a tertiary referral hospital.

**Materials and methods:**

A prospective study was undertaken between May and October 2017 of 52 children who presented with suspected appendicitis and were referred for an abdominal ultrasound. All patients included in this study received both an abdominal ultrasound and five-sequence MRI consisting of axial and coronal gradient echo T2 scans, fat-saturated SSFSE and a diffusion-weighted scan. Participants were randomised into groups of MRI with breath-holds or MRI with free breathing. A patient satisfaction survey was also carried out. Histopathology findings, where available, were used as a gold standard for the purposes of data analysis. Statistical analysis was performed, and *p* values < 0.05 were considered statistically significant.

**Results:**

Ultrasound had a sensitivity and specificity of 25% and 92.9%, respectively. MRI with breath-hold had a sensitivity and specificity of 81.8% and 66.7%, respectively, whilst MRI with free breathing was superior with sensitivity and specificity of 92.3% and 84.2%, respectively. MRI with free breathing was also more time efficient (*p* < 0.0001). Group statistics were comparable (*p* < 0.05).

**Conclusions:**

The use of fast acquisition MRI protocols, particularly free breathing sequences, for patients admitted with suspected appendicitis can result in faster diagnosis, treatment and discharge. It also has a statistically significant diagnostic advantage over ultrasound. Additionally, the higher specificity of MR can reduce the number of negative appendectomies performed in tertiary centres.

## Key points

- MRI offers an alternative imaging option in cases of suspected acute appendicitis in children.
- Fast acquisition MRI protocols are capable of diagnosing or excluding acute appendicitis.
- Free breathing MRI imaging sequences have improved diagnostic accuracy compared with breath-hold sequences.

## Introduction

Abdominal pain is a common presenting complaint among children. It is the third most common reason for emergency department (ED) attendances among females, and the sixth most common among males, under 15 years of age in the United States (US) [1]. There are many causes of acute abdominal pain; acute appendicitis represents the most common paediatric surgical complaint in the US [2]. Prior to the widespread use of diagnostic imaging, the diagnosis of acute appendicitis was primarily made on clinical grounds. This practice was associated with resection of a normal appendix (negative appendectomy) in approximately 14% of cases proceeding to surgery [3]. Medical imaging has improved this practice.

Classic imaging findings in acute appendicitis, across a range of modalities, include the presence of a dilated, thick-walled, blind-ending, tubular structure with a diameter in excess of 6 mm [4]. Periappendiceal inflammation and prominent mucosal enhancement, with or without an appendicolith, are also suggestive. On MRI, an acutely inflamed appendix is further suspected when the appendiceal wall is noted to be more T1-hypointense and T2-hyperintense [5].

Ultrasound was adopted for the investigation of acute appendicitis in children during the mid 1980s and remained the imaging investigation of choice in the US through the mid 1990s [6]. Computed tomography (CT) gradually replaced ultrasound as the primary assessment of suspected acute appendicitis in the US [7]. The widespread use of CT imaging has contributed to a reduction in the rate of negative appendectomy from as high as 23% to as low as 1.7% [8].

More recently, MRI has been evaluated for the investigation of suspected acute appendicitis in children. The performance of MRI in the paediatric setting needs to be tailored towards short examination times. Therefore, fast acquisition T2 sequences in multiple planes (e.g. half acquisition single shot fast spin echo (SSFSE)) are frequently used [9]. Contrast administration tends to be omitted from paediatric protocols which may decrease confidence of interpretation, though in the majority of cases this is typically within acceptable limits [10]. Sedation is also generally not administered, and equivalent sensitivity and specificity compared with CT have been reported in a matched cohort of non-sedated children [9]. In addition to considerations related to paediatric patient cooperation and tolerance, time in the MR scanner is also an important factor, which can affect the adoption of MRI for assessment of acute appendicitis.

In order to optimise scanner time, sequences need to be chosen that are short to acquire and have good sensitivity for acute appendicitis. The use of free breathing protocols has facilitated diagnostic imaging in under 10 min [11]. It is unclear how free breathing sequences compare with breath-hold sequences for the assessment of acute appendicitis. Diffusion-weighted imaging (DWI) has also been shown to aid in the diagnostic accuracy of MRI for acute appendicitis, though the added time required for imaging may not be justified [12]. The advent of three-Tesla (3-T) MRI imaging also presents the opportunity to perform imaging in a more time-efficient manner.

The purpose of the present paper was to compare the diagnostic accuracy of breath-hold and free breathing 3-T MRI sequences for the investigation of acute appendicitis in children, and also to assess whether DWI yields useful additional information. Our secondary goal was to compare the accuracy of MRI with departmental ultrasound in a general hospital setting.

## Materials and methods

Regional ethical approval was obtained prior to this prospective, randomised, controlled cohort study. Inclusion criteria consisted of any patient aged between 5 and 16 years (inclusive) presenting to the ED with symptoms and signs suggestive of acute appendicitis, requiring ultrasound assessment for the investigation of suspected appendicitis. The on-duty surgical team reviewed all patients prior to the referral for ultrasonography. Patients with a history of previous abdominal surgery and abdominal malignancy, or who had behavioural/cognitive disorders that would preclude the use of an MRI scanner were excluded. Patient recruitment and informed consent was obtained prior to the ultrasound for all subjects.

The on-duty consultant radiologist and radiology resident performed all ultrasound examinations. Ultrasound examination was performed using a graded compression technique with a high-frequency probe (11 MHz) (General Electric, WI, USA; ML6-15-D) using standard abdominal pre-sets. The ultrasound report was made available immediately following assessment for review by the referring physician.

MR imaging was performed using a 3-T MRI scanner (General Electric, WI, USA; 750W, software version DV25.1_1649a) for all recruited patients. All patients were scanned in a fasting state with a minimum fasting time of 4 h. Parents or guardians completed a standard pre-MRI questionnaire, and the patients were then placed supine on the scanner gantry. A standard body coil (GEM anterior array coil) was used, and a feet-first orientation was adopted. Three patients were imaged at the beginning of the study in order to determine the study protocol. Data from these scans was not included in the final results.

The study group was assigned sequentially into two cohorts; the first 20 consecutive patients were scanned with breath-hold MRI sequences, whilst the second half of the patient group (*n* = 32) were assigned to the free breathing group.

MRI protocol consisted of axial and coronal T2 single shot fast spin echo (SSFSE) with and without fat saturation (FS) followed by axial diffusion-weighted imaging (DWI) (Table 1). Axial scans extended from the right renal hilum to the equator of the femoral heads. Coronal scans included the full abdomen with a field of view including the upper pole of the left kidney to the pubic symphysis. DWI was performed for all patients. Diaphragmatic triggering was used for acquisition and imaging performed over the same range as the axial T2 scans. The diagnostic utility of DWI was compared with the T2 sequences that were acquired.

**Table 1:** Imaging protocol parameters for MRI of suspected acute appendicitis

Parameter	2D SSFSE with fat suppression		2D SSFSE without fat suppression		2D DWI with fat suppression
	Axial	Coronal	Axial	Coronal	Axial
**TR (ms)**	1388–1701	1120–1720	1190–1706	1373–1706	4000/7500
**TE (ms)**	88.06–102.8	87.12–92.45	88.06–92.42	87.12–92.45	68.9–71.2
**FOV (cm)**	40.0	38.0	40.0	38.0	40.0
**Matrix**	384 × 160	325 × 256	352 × 256	352 × 256	96 × 28
**Bandwidth (kHz)**	83.33	83.33	83.33	83.33	250
**Slice thickness (mm)**	3.0	3.0	3.0	3.0	5.5
**Slice spacing (mm)**	0.3	0.3	0.3	0.3	0.5
**No. of signal averages**	1	1	1	1	1

Images were reviewed on a picture archiving and communication system (Impax 6.5.3; Agfa Healthcare, Morstel, Belgium) on a three-megapixel monitor (Barco Model MFGD 2621). The MRI examinations were read at the time of acquisition by the paper co-authors and reports issued to the requesting physician. For the purposes of the paper, the MRI scans were read separately (after completion of patient recruitment) by two fellowship-trained radiologists with 23 years (A) and 14 years (B) of experience, respectively. DWI was assessed separately by radiologist B and a radiology trainee with 1 year’s experience.

All research reads were blinded to clinical, ultrasound and histopathology information.

Histopathology findings, where available, were used as a gold standard for the purposes of data analysis. Patients who were treated non-surgically were followed for 6 months to assess for recurrent symptoms.

To assess the diagnostic accuracy of imaging, results were characterised based on a modification of a previously published format; a four-point system was used to determine the presence of acute appendicitis [13] (Table 2; U = ultrasound score, M = MRI score). Across both modalities, a score of four indicates appendicitis; this score is only assigned if the appendix is definitively seen and noted to be abnormal, with thickening and/or evidence of inflammation.

**Table 2:** Modified scoring system for classification of both MR and ultrasound radiological findings, in cases of suspected acute appendicitis. Values range from 1 to 4; score of 1 indicates no appendicitis, score of 4 indicates appendicitis

U1/M1	Normal appendix visualised fully; no appendicitis
U2/M2	Equivocal but leaning towards no appendicitis (i.e. no appendix seen, with no secondary signs of appendicitis *OR* appendix seen and indeterminate features but not thought to be appendicitis *OR* appendix partially seen and thought to be normal)
U3/M3	Equivocal but leaning towards appendicitis (i.e. no appendix seen, but suggestive secondary signs of appendicitis such as echogenic or hyperaemic fat on ultrasound or mesenteric fat changes on MR *OR* appendix seen with indeterminate features suggesting appendicitis)
U4/M4	Abnormal appendix visualised; appendicitis

*U* ultrasound score, *M* MRI score

Scanning duration was calculated from the time of initial image acquisition to that of the final image acquired. Ultrasound times only included time on the couch. Circumstances where patients were sent for further bladder filling etc. were not included in the overall assessment of scan duration. MRI studies were acquired at a single visit, and the time from the first to the final sequence acquisition was recorded.

Patient records were reviewed to record the duration of symptoms, biochemical indices of inflammation (white cell count (WCC), C-reactive protein (CRP)) and length of inpatient hospital stay.

Each patient was asked to rate their experience based on the options provided on a Likert-scale scoring card following completion of the ultrasound and MRI scans.

Results were presented using Microsoft Office Excel 2010 (Microsoft Corporation, CA, USA), and statistical analysis was performed using GraphPad Prism (GraphPad Software Incorporated, San Diego, USA) and the Statistical Package for the Social Sciences (SPSS) version 24 (IBM, Chicago, IL, USA). Receiver operator curves were created using SPSS to assess the accuracy of MRI and ultrasound for the diagnosis of acute appendicitis. The distribution of variable data was assessed using a D’Agostino-Pearson omnibus normality test. Wilcoxon matched-pairs signed rank test was used to compare non-parametric data regarding scan times between ultrasound and MRI for each cohort. Mann-Whitney test was used to compare scan times between the free breathing and breath-hold MRI cohorts. Continuous variables were tested for normality using the Kolmogorov-Smirnov test. Comparisons between the two patient groups were performed using an independent *t* test or the Mann-Whitney *U* test (as appropriate) for continuous data. *p* values less than 0.05 were considered to be statistically significant. Non-Gaussian variable data was expressed in the form of median with interquartile range (IQR).

## Results

Fifty-two patients (18 males, 34 females) who presented to the ED between May and August 2017 and who met the inclusion criteria were recruited prospectively and included in the study.

The mean age of study participants was 11 ± 2 years (range 5–16 years). The mean body mass index (BMI) measured 20.14 ± 3.2. There was no statistically significant difference in age or BMI between the breath-hold and free breathing study groups. Symptom duration, inflammatory indices and length of hospital stay were similar between groups (Table 3).

**Table 3:** Comparison of clinical parameters of subjects from both study groups. There was no statistically significant difference between groups

Characteristic	Free breathing group	Breath-hold group	*p* value
Symptom duration (days)	1.9	2.2	0.537^a^
WCC (cells/L)	18.5	13.4	0.494^a^
CRP (mg/L)	12.4	10.5	0.212^a^
Length of inpatient hospital stay (days)	2.9	3.1	0.748^a^

^a^Independent *t* test

**Fig. 1:**
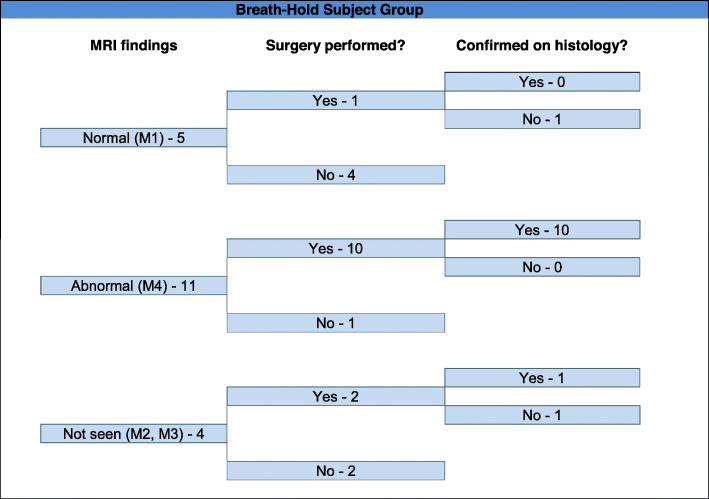
Data from the patient cohort (*n* = 20) assigned to MRI with breath-hold. The appendix was demonstrated to be normal in five and abnormal in eleven cases. All patients with an abnormal appendix who proceeded to surgery had appendicitis confirmed

**Fig. 2:**
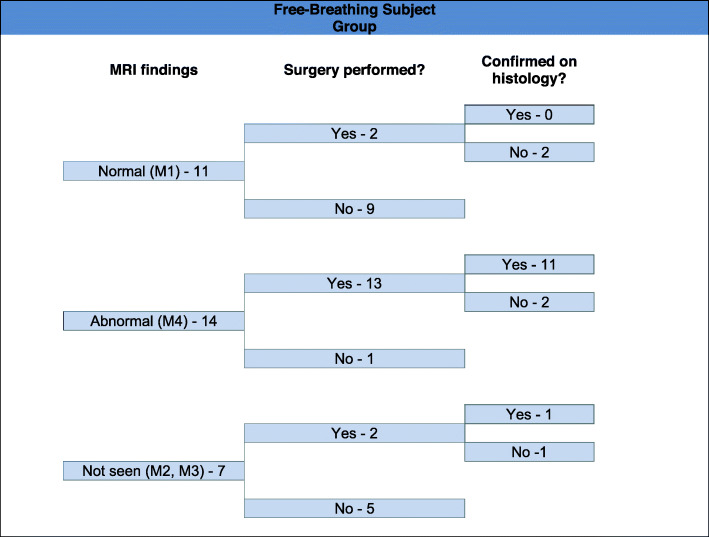
Data from the patient cohort (*n* = 32) assigned to MRI with free breathing. The appendix was demonstrated to be normal in eleven and abnormal in fourteen cases. Thirteen abnormal cases proceeded to surgery; eleven of these had appendicitis confirmed. One case with appearances of acute appendicitis (M4) on MRI had luminal enterobius vermicularis confirmed histologically, without acute appendicitis

**Fig. 3:**
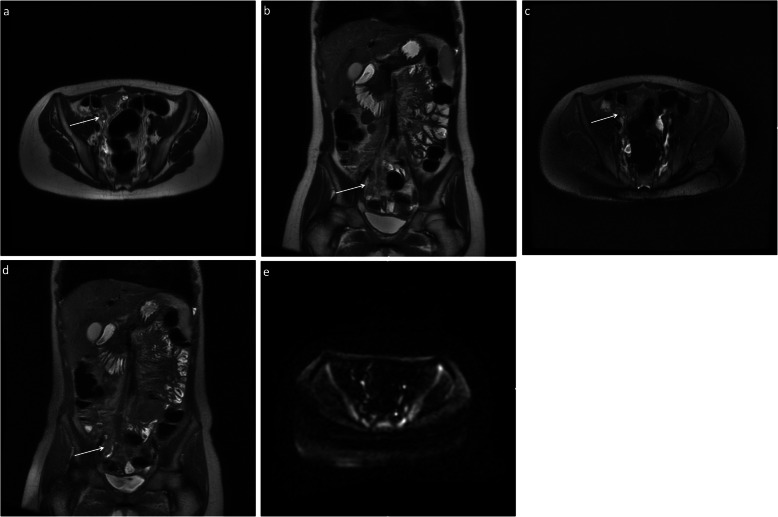
Five-sequence breath-hold MRI on a 12-year-old female patient with acute appendicitis (M4), confirmed histologically. The appendix was not demonstrated on ultrasound. There is thickening of the appendix and periappendiceal fat stranding (arrows). There was no increased signal on DWI. Axial (**a**) and coronal (**b**) T2-weighted without fat saturation, respectively; axial (**c**) and coronal (**d**) T2-weighted with fat saturation, respectively; axial (**e**) DWI B = 1400

**Fig. 4:**
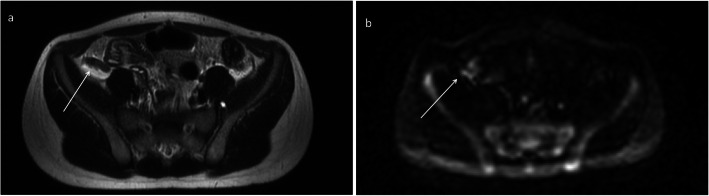
Five-sequence free breathing MRI on an 8-year-old male patient with radiological findings suggestive of acute appendicitis (M4). The appendix was not demonstrated on ultrasound. There is thickening of the appendix and periappendiceal fat stranding (arrows). There was increased T2 signal on DWI. Luminal enterobius vermicularis confirmed histologically; however, there was no acute appendicits present. Axial (**a**) T2-weighted without fat saturation and (**b**) DWI B = 1400 MRI images are presented, respectively

**Fig. 5:**
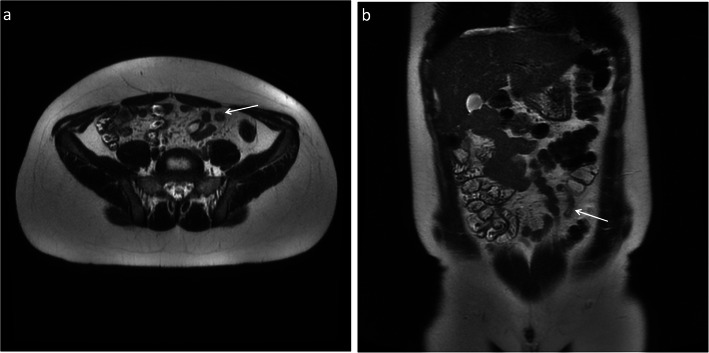
Midgut malrotation; T2-weighted MRI without fat saturation in a 12-year-old male patient demonstrating the appendix situated to the left of midline, deep to the rectus abdominis muscle. It is thickened near the tip measuring 9 mm with surrounding inflammatory fat stranding (arrows). Axial (**a**) and coronal (**b**) T2-weighted MRI images are presented, respectively

Pathology results were obtained on all 30 patients who underwent surgery, and 23 abnormal appendixes (11 in the breath-hold group (Fig. 1) and 12 in the free breathing group (Fig. 2)) were confirmed (Fig. 3). Seven patients had a normal appendix at surgery; one of these was found to have a corpus luteal cyst, demonstrated on both ultrasound and MRI. None of the patients who were discharged without surgery re-presented with abdominal pain. Three patients who underwent surgery were found to have luminal enterobius vermicularis but no signs of acute inflammation on pathology. One of these patients had a false positive finding of an abnormal appendix on MRI (Fig. 4). There was one case of acute appendicitis in a patient with incidental midgut malrotation (Fig. 5).

### Ultrasound

**Fig. 6:**
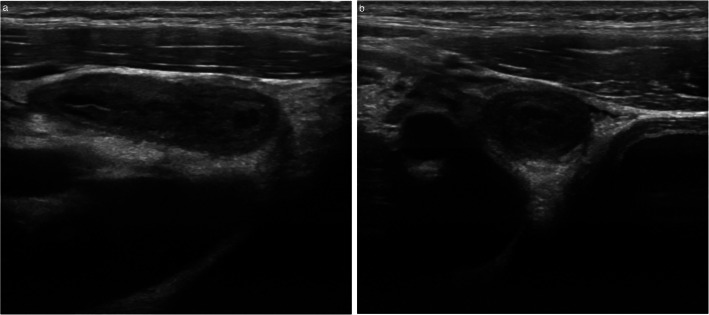
Ultrasound findings in a 14-year-old male patient with acute appendicitis (U4), confirmed histologically. The appendix is dilated, measuring up to 1 cm in short axis diameter with mural thickening. Longitudinal (**a**) and transverse (**b**) ultrasound images are presented

A normal appearing appendix was demonstrated in two of the 52 patients who were scanned (U1 = 2) (Table 4). Of those in whom the appendix was not seen or only partially seen, no secondary inflammatory signs were noted in 42 patients (U2 = 42). Signs of inflammation without visualisation of the appendix were found in two patients (U3 = 2), and an abnormal appendix was definitively demonstrated in six patients (U4 = 6) (Fig. 6). A receiver operator characteristic (ROC) curve was created, and the area under the curve (AUC) for ultrasound was 0.59 ((asymptomatic 95% confidence interval (CI) 0.428–7.45), (asymptomatic *p* value = 0.29)). The sensitivity and specificity were 25% and 92.9%, respectively.

**Table 4:** Distribution of U scores following ultrasound assessment for suspected acute appendicitis. Six patients were diagnosed with acute appendicitis

Ultrasound score	Number of patients
U1	2
U2	42
U3	2
U4	6

### MRI

**Fig. 7:**
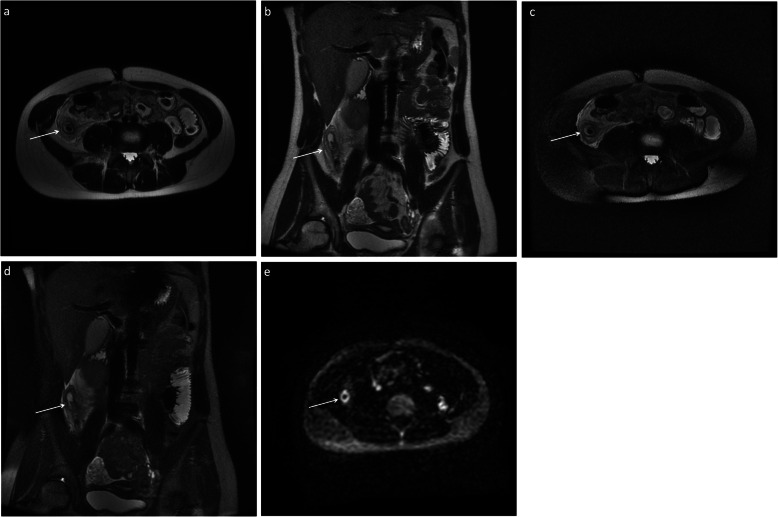
Five-sequence free breathing MRI on a 15-year-old female patient with acute appendicitis (M4), confirmed histologically. The appendix was not demonstrated on ultrasound. There is thickening of the appendix and periappendiceal fat stranding (arrows). There was increased signal on DWI. Axial (**a**) and coronal (**b**) T2-weighted without fat saturation, respectively; axial (**c**) and coronal (**d**) T2-weighted with fat saturation, respectively; axial (**e**) DWI B = 1400

The MRI with breath-hold protocol demonstrated a normal appendix in five patients (M1 = 5). MRI was unable to identify the appendix in an otherwise normal appearing abdomen in three patients (M2 = 3). Signs of inflammation without visualisation of the appendix occurred in only one patient (M3 = 1). A thickened and/or inflamed appendix was demonstrated in 11 patients (M4 = 11) (Fig. 7).

On the free breathing MRI scans, a normal appearing appendix was demonstrated in 11 patients (M1 = 11). The appendix was not demonstrated in an otherwise normal appearing abdomen in six patients (M2 = 6). An abnormal appendix was demonstrated in 14 patients (M4 = 14), and signs of inflammation without visualisation of the appendix occurred only once (M3 = 1).

The AUC for all MRI scans was 0.88 (95% CI 0.779–0.975) (*p* value < 0.001). The sensitivity and specificity were 82.5% and 72.6%, respectively, with positive and negative predictive values of 82.84 and 78.83, respectively.

When comparing the accuracy of breath-hold MRI and free breathing MRI, the AUC for breath-hold MRI was 0.78 (95% CI 0.552–1.000) (*p* value = 0.04) with a sensitivity and specificity of 81.8% and 66.7%, respectively. The AUC for free breathing MRI measured 0.93 (95% CI 0.835–1.000) (*p* value < 0.001) with associated sensitivity and specificity values of 92.3% and 84.2%, respectively.

The AUC for DWI alone was 0.76 (95% CI 0.61–0.89) (*p* value = 0.002), with a sensitivity and specificity of 75% and 84%, respectively. It was found that DWI did not affect the determination of the score allocated on MRI.

### Scan duration assessment

Median duration of ultrasound examination in patients examined with breath-hold MRI was 17 min, 40 s (IQR 13 min, 17 s to 24 min, 52 s), and for patients imaged with free breathing MRI, it was 17 min, 12 s (IQR 11 min, 20 s to 20 min, 48 s).

The median scan duration for the breath-hold MRI was 17 min, 24 s (IQR 15 min, 32 s to 19 min, 50 s). The median scan duration for the free breathing MRI was 12 min, 18.5 s (IQR 11 min, 19 s to 15 min, 12 s).

The free breathing MR scans were significantly faster to acquire than MRI with breath-holding techniques (*p* < 0.0001), and they were also significantly faster (although to a lesser extent) than the corresponding ultrasound scans for the same cohort (*p* = 0.018). There was no significant time saving advantage when comparing breath-hold MRI studies with its ultrasound cohort (*p* = 0.55).

### Patient satisfaction

Forty-three of 52 patients responded to the satisfaction questionnaire. A score of 1 indicated the least satisfaction, whilst a score of 5 indicated the most satisfaction. The median score for both the MRI and ultrasound was 4 (*p* = 0.7).

## Discussion

The goal of imaging in paediatric patients with suspected acute appendicitis is to either correctly diagnose acute appendicitis, or correctly identify a normal appendix and consequently reduce the occurrence of unnecessary surgeries.

Ultrasound remains widely used as a first-line imaging tool for suspected acute appendicitis, particularly outside the US. Ultrasound detection of acute appendicitis is operator dependent however, and a multicentre study found that in centres where ultrasound was not routinely used for investigation of acute appendicitis in children, the sensitivity for acute appendicitis can drop to approximately 50%, and even as low as 35% [14]. Other factors such as childhood obesity can reduce the accuracy of ultrasound assessment, especially in patients with a low pre-test probability for acute appendicitis [15].

Whilst ultrasound provides the facility to scan without a requirement for sedation or to remain absolutely still [11], a meta-analysis comparing CT with ultrasound for the diagnosis of acute appendicitis in children showed superior sensitivity and specificity with CT compared with ultrasound (88% and 94% for ultrasound versus 94% and 95% for CT) [16], and a single-centre study has reported that the normal appendix could be seen in 82% of children [13]. CT use for paediatric imaging is limited due to concerns regarding ionising radiation exposure. Additionally, the relative lack of visceral adipose tissue in children can hinder CT interpretation [9, 10]. Therefore, there is increasing interest in the performance of MRI for the assessment of acute abdominal pain in paediatric patients.

There is no consensus regarding optimal imaging sequences at present. If MRI is to succeed for the examination of appendicitis, the study needs to be quick to perform and agreeable to the patient. The present paper compared free breathing with breath-hold imaging sequences, and compared diagnostic yield and patient satisfaction with ultrasound, which represents the first-line imaging modality in many centres.

The present results indicate that fast acquisition T2 and T2 fat-saturated scans acquired in the axial and coronal planes are sufficient in the vast majority of cases to enable the radiologist to correctly diagnose or exclude acute appendicitis. Interestingly, one of the consultants performing the reading of scans anecdotally reported that the non-fat-saturated T2 sequence allowed for better localisation of the abnormal appendix. However, the fat-saturated versus non-fat-saturated images were not compared in a blinded manner, and so true efficacy of one over another cannot be determined from the current study.

DWI did not influence the outcome of the scan results in the present paper. This contrasts to a prior study demonstrating the improved diagnostic accuracy with 1.5-T MRI performed using DWI and non-contrast sequences [12]. At 3.0 T, DWI benefits from increased signal to noise ratio, though conversely, increased magnetic susceptibility often contributes to a loss of image quality [17]. Whilst an acute pathology such as acute appendicitis returns high signal intensity on fat-suppressed diffusion-weighted images due to a combination of restricted diffusion and oedema [18], we are unaware of any study which has directly compared the diagnostic capability of DWI for 1.5-T or 3.0-T MR imaging in acute appendicitis. DWI added at least 2 min (and in some cases four minutes) of scan time to the total scan duration. Curtailing MRI table time still further in order to minimise patient discomfort could be achieved by omitting this sequence.

The type of breathing manoeuvre employed during image acquisition also affects scanner time [15]. The present paper confirms that breath-hold sequences take significantly longer than free breathing studies to perform (by approximately five minutes). In an emergency setting, MR imaging protocols can be broadly grouped into either free breathing protocols or breath-hold protocols. Whilst free breathing protocols are preferable for individuals unable to hold their breath for longer than 20 s, breath-hold sequences have a role in the delineation of haemorrhagic collections [19]. Our results demonstrate that MRI accuracy was improved when gentle free breathing sequences were used during scan acquisition. This likely reflects a relative decrease in respiratory motion artefact, as younger children, and those in significant pain, may have been unable to co-operate fully with breath-hold instructions. It was noted that the position at which the diaphragm was held during breath-holds differed from one acquisition to the next, resulting in artefact which may have affected image quality.

Our MRI department provides a scanning service during extended daytime hours, 7 days per week; outpatient MRI scans are routinely scheduled during weekend daytime hours. Overnight, the MRI scanner is available for emergency neurosurgical referrals. We are confident that these operating hours did not contribute to a delay in the transfer of patients with suspected acute appendicitis to theatre for surgery. The above MRI protocol necessitated no contrast administration and had a relatively short associated scan duration. Furthermore, our MRI department has dedicated research time assigned. Consequently, radiographers were very accommodating in scheduling MRI examinations at short notice.

The low yield of ultrasound should be acknowledged. This is greatly affected by the criteria used to diagnose acute appendicitis. In the author’s institution, acute appendicitis is only diagnosed in suspected cases when the appendix can be definitively demonstrated on ultrasound, and is confirmed to be abnormal (U4; Table 2); the appendix may be thickened, or demonstrate evidence of inflammation. We define a thickened appendix as one with a diameter in excess of 6 mm. Widely referenced abnormalities on ultrasound [20–24], which we accept as evidence of inflammation in an abnormal appendix, include a non-compressible appendix, a hypoechoic fluid-filled lumen, a hyperechoic mucosa/submucosa and hypervascularity in the early stages. Ultrasound features that are classified as indeterminate, and therefore only suggestive of appendicitis (U3; Table 2), include free fluid surrounding the appendix, focal ultrasound probe tenderness, increased echogenicity of local mesenteric fat and enlarged local mesenteric lymph nodes.

Operator variation could have an influence on the results of the paper; however, all staff involved in patient imaging were either paediatric or abdominal fellowship trained and proficient in the use of ultrasound for this purpose. This study was not performed in a dedicated ultrasound or paediatric imaging centre which improved the sensitivity of ultrasound for the detection of acute appendicitis from 71 to 88% in one study [25]. In addition, many of the examinations in the present paper were performed out of standard weekday working hours, on an on-call basis, which could also affect results. A wide variability in the sensitivity of ultrasound for the detection (between 44 and 100%) of acute appendicitis in adults has been reported in the literature [26]. The low sensitivity of ultrasound in our centre was one of the reasons why alternative, more reliable, methods for the diagnosis of acute appendicitis were investigated.

Median duration of ultrasound assessment, for all patients, was just over 17 min. The duration of ultrasound imaging is seldom reported in the literature; when evaluated, it is often measured in terms of patient waiting times, transfer or turnaround times [27, 28]. The duration of the ultrasound imaging component of the examination was recorded as this was deemed most comparable with the on-table component of the MRI examination as regards length of time that patients in the present paper underwent investigation. One aspect of the purpose of the present paper was to assess whether ultrasound or a truncated MRI was preferred by patients; duration of the actual imaging component was therefore deemed important for analysis. On questioning, patients did not express a preference for ultrasound over MRI. Examination of the reasons for their choices was beyond the scope of this study, though scan duration does have an effect on the ability of children to tolerate MRI [29].

An assessment of the financial implications of performing MRI routinely for paediatric patients presenting to the ED with abdominal pain was not performed. Recently published work maintains that ultrasound assessment is still the most time-efficient and cost-effective investigation for children with suspected acute appendicitis [28]. However, a German study, in which MRI was used to assess 52 equivocal cases of suspected appendicitis, obviated the need for surgery in three patients, resulting in a net cost saving of €3453 per patient [30]. The use of advanced imaging technologies may have significant cost implications for healthcare providers worldwide. However, efficient use in a targeted manner could ameliorate the overall economic burden for the health service, improving patient outcomes and delivering a more rapid journey through the system.

The superiority of MRI as the first-line investigation for suspected appendicitis was shown in a recent single-centre retrospective review of 402 paedatric patients [31]. This study reported an appendix visualisation rate of 86.8% and sensitivity of 97.9% for non-contrast-enhanced MRI; an alternate diagnosis was provided in 113 of 304 patients negative for appendicitis. These findings echo the current paper confirming information gained from MRI as being comparable or better than that of ultrasound for the diagnosis of acute appendicitis.

The experience and findings of conducting the current paper have positively impacted on imaging assessment for suspected acute appendicitis within our institution. Ultrasound remains the first-line imaging investigation for patients with suspected acute appendicitis. CT examination is used less frequently in cases where there is diagnostic dilemma and instead there is a much lower threshold for performing MRI than prior to performance of this study. Radiographers and radiologists are satisfied that diagnostic imaging can be acquired and are therefore more likely to recommend MRI. Surgeons are aware that additional assessment by means of MRI can be performed when required. In addition, the MRI protocol used in the present paper has been used for expedient imaging (to avoid prolonged inferior vena compression) in pregnant patients with suspected acute appendicitis where ultrasound has not been diagnostic.

The false positive MRI result involving enterobius vermicularis should also be acknowledged. The appendix specimen in this case showed no histopathological evidence of inflammation, yet it was diagnosed as acute appendicitis on the MRI scan due to thickening of the appendix. This suggests that the protocol used in the current study may not be sufficiently sensitive for this specific pathology. However, the limited number of cases in our study is inadequate to draw firm conclusions.

### Conclusion

The present paper demonstrates that 3-T MRI for the assessment of acute appendicitis can be successfully performed in a paediatric population with improved accuracy compared with ultrasound. This is mainly due to the ability to exclude acute appendicitis once a normal appendix has been demonstrated. In the era of increasing childhood obesity, MRI can be a useful adjunct to ultrasound in selected cases, but false positive findings do occur. Free breathing imaging sequences were shown to be significantly faster to perform and had superior diagnostic accuracy compared with breath-hold sequences. DWI did not improve diagnostic performance.

## Data Availability

The majority of datasets analysed and generated during this study are included in this published study; on reasonable request, further datasets are available from the corresponding author.

## Abbreviations

AUC: Area under the curve
BMI: Body mass index
CI: Confidence interval
CRP: C-reactive protein
CT: Computed tomography
DWI: Diffusion-weighted imaging
ED: Emergency department
FS: Fat saturation
IQR: Interquartile range
MR: Magnetic resonance
MRI: Magnetic resonance imaging
ROC: Receiver operator characteristic
SPSS: Statistical Package for the Social Sciences
SSFSE: Single shot fast spin echo
T: Tesla
US: United States
WCC: White cell count
